# Photonic Methods to Enhance Fluorescence Correlation Spectroscopy and Single Molecule Fluorescence Detection

**DOI:** 10.3390/ijms11010206

**Published:** 2010-01-13

**Authors:** Jérome Wenger, Hervé Rigneault

**Affiliations:** Institut Fresnel, Aix-Marseille Université, CNRS, Ecole Centrale Marseille, Domaine Universitaire de Saint-Jérôme, 13397 Marseille cedex 20, France

**Keywords:** single molecule, fluorescence correlation spectroscopy FCS, nanophotonics, biophotonics

## Abstract

Recent advances in nanophotonics open the way for promising applications towards efficient single molecule fluorescence analysis. In this review, we discuss how photonic methods bring innovative solutions for two essential questions: how to detect a single molecule in a highly concentrated solution, and how to enhance the faint optical signal emitted per molecule? The focus is set primarily on the widely used technique of fluorescence correlation spectroscopy (FCS), yet the discussion can be extended to other single molecule detection methods.

## 1. Introduction

The capacity to detect and analyze optical signals emitted from single molecules is a key issue in nanosciences, especially for biological applications. Data from a single molecule may reveal information hidden by ensemble measurements, such as variances in kinetic rates, memory effects, or transient states [1]. Observing a single or few dye molecules enables the study of dynamics and characteristics of the sample without actually disturbing their equilibrium. Therefore, efficiently detecting a single molecule has become a major goal with applications in chemical, biochemical and biophysical analysis.

Among the large number of methods that have been developed to investigate single molecules [2], fluorescence spectroscopy plays the largest role today. Fluorescence bears a high intrinsic optical efficiency, and provides information about the molecular environment and structure in many different ways: brightness, lifetime, anisotropy or spectrum.

To analyse the fluorescence signal from a single molecule, fluorescence correlation spectroscopy (FCS) is a powerful and versatile method [3, 4]. FCS is based on the statistical analysis of the temporal fluctuations affecting the fluorescence intensity. It can in principle provide information about any molecular process that induces a change in the fluorescence intensity. For instance, fluctuations occur when molecules diffuse in and out of an observation volume, or when reaction kinetics or conformational changes induce a change in the fluorescence brightness. Applications include determining translational and rotational diffusion, molecular concentrations, chemical kinetics, and binding reactions [5].

**Figure 1.**
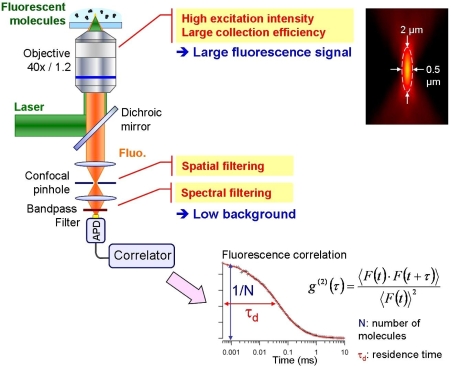
State-of-the-art for single molecule detection in solution based on a confocal microscope and fluorescence correlation analysis. The inset shows the size of the typical analysis volume.

A critical issue in FCS as in any single molecule experiment is to discriminate the (weak) relevant signal from the noise, which requires simultaneously high fluorescence count rates per molecule and low background. Therefore, FCS is commonly implemented on a confocal microscope with a high-end immersion objective, providing high resolution and large numerical aperture (see Figure 1). Despite its extreme sensitivity, this approach remains limited by the phenomenon of optical diffraction.

Optical diffraction limits confocal microscopy in two ways. First, it restricts the analysis volume to rather large values of typically 0.5 femtoliter (= 0.5 *μ*m^3^). This amounts to a useful concentration in the nanomolar range in order to isolate only a few molecules, which comes in contradiction to the concentrations in the micro to the millimolar range typically found in living cells. Second, optical diffraction restricts confocal microscopy to relatively low detection rates per molecule. This limits the choice of fluorescence markers to relatively bright species, and imposes long integration times.

**Figure 2.**
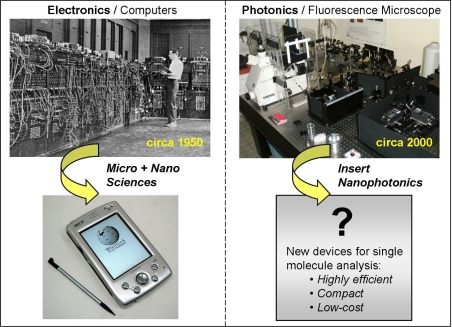
As in computer engineering, nanosciences enable major breakthroughs in designing novel microscope tools and devices (computers pictures courtesy of Wikipedia.org).

Recent advances in nanophotonics and fluorescence microscopy offer new solutions to overcome the limits imposed by diffraction [6–8]. Drawing a parallel with computer engineering (Figure 2), nanosciences enable major breakthroughs in designing novel photonic tools for single molecule analysis. The observation volume can be reduced by several orders of magnitude below the diffraction-limited confocal volume, while the detected fluorescence rate per molecule can be enhanced more than ten times. The photonic environment can affect the fluorescence emission in three ways: (i) by locally enhancing the excitation intensity, (ii) by increasing the emitter’s radiative rate and quantum efficiency, and (iii) by modifying its radiation pattern, towards a higher emission directionality to the detectors. A major goal is to tailor the electromagnetic environment surrounding the molecules, so as to simultaneously enhance the collected fluorescence and decrease the detection volume to reduce background noise and enlarge the practicable domain of concentrations for single-molecule analysis.

In this contribution, we focus on two fundamental questions that bear highly practical applications: how to detect one single molecule in a highly concentrated solution, and how to enhance the faint optical signal emitted per molecule? Both key parameters of volume reduction and fluorescence rate enhancement will be discussed. The focus is set primarily on the widely used technique of fluorescence correlation spectroscopy (FCS), yet the discussion can be extended to other single molecule detection methods. We divide the different strategies into two main areas. The first one takes advantages of shaping the laser excitation beam in an unconventional manner (Section 2), while the second introduces photonic structures to overcome the diffraction limit (Section 3).

## 2. Improved Single Molecule Fluorescence Detection by Structuring the Laser Excitation Beam

**Figure 3.**
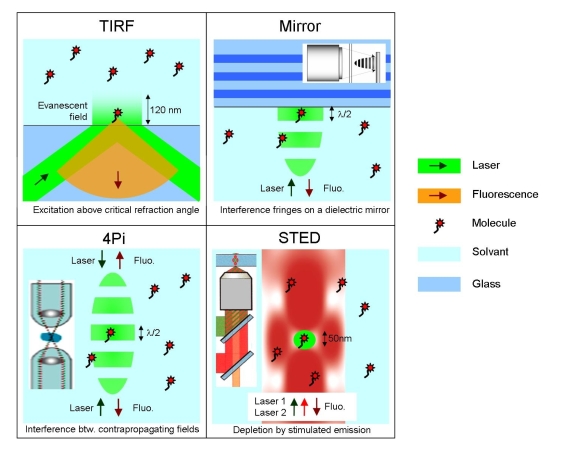
Designs for improved single molecule fluorescence detection by structuring the laser excitation beam; see text for details.

To improve single molecule fluorescence detection, several optical methods have been suggested and tested in light microscopy. The methods regrouped in this section share the fact that they primarily focus on modifying the laser excitation beam to further confine light on the nanoscale and improve the spatial resolution. Figure 3 describes the different methods of this section, the line at the bottom of each picture summarizes the main physical concept used.

### 2.1. Total Internal Reflection Fluorescence Microscopy: TIRF

This system takes advantage of a total internal reflection fluorescence (TIRF) microscopy setup. Fluorescent molecules diffusing above the upper interface of the TIRF prism or objective are excited by an evanescent wave generated by total internal reflection at the solid/liquid interface [9]. The shallow evanescent field in the liquid elegantly defines an analysis volume that is strongly reduced along the longitudinal direction. Its longitudinal extend typically amounts to ~ λ/6, which offers a reduction of 10 compared to conventional confocal microscopes. An important feature for this technique is that still a confocal pinhole conjugated to the object plane has to be used in order to reduce the lateral dimensions of the fluorescence analysis volume [10]. TIRF-FCS offers an excellent axial confinement, yet it does not provide lateral confinement of the excitation profile and a pinhole in the image plane is needed to reduce the lateral extension of the detection profile. Therefore, out-of-focus photobleaching can be a major issue in TIRF-FCS, leading to a depletion of fluorophores and limiting the accuracy of FCS measurements.

Originally introduced in 1981 [9], this technique can now be combined with commercially available microscope objectives for TIRF imaging [10]. This tool can be used as efficiently for analyzing molecules diffusion in solution or in a lipid membrane [11], and is also compatible with multi-dye fluorescence detection [13].

Lastly, it can be combined with a thin metal layer for specific excitation of surface plasmon modes, which offers further background noise reduction and longitudinal confinement [12].

### 2.2. Fluorescence Detection on a Mirror

Single molecule detection in solution is tightly bound to the implementation of confocal microscopy, as depicted on Figure 1. An elegant way to reduce the confocal analysis volume and enhance the fluorescence rate emitted per molecule takes advantage of a dielectric mirror set at the focal point of the excitation beam (see Figure 3). The mirror affects both the laser excitation intensity and pattern, and the collection of the emitted fluorescence. The coherent excitation beam, which is reflected, produces an interference pattern along the optical axis with an interfringe spacing of λ/2*n*, where λ is the excitation wavelength and *n* is the medium refractive index.

Two important effects occur when the confocal detection volume is located on the mirrors surface [14, 15]. First, interference fringes in the excitation beam give rise to a new characteristic time in the fluorescence correlation function. This new time is found to be independent of the transverse excitation fields beam waist and permits accurate measurement of diffusion coefficients without any a priori knowledge of the confocal volume geometry. Second, the count rate per emitter is significantly enhanced owing to control of spontaneous emission and enhancement of the excitation field, with a gain up to four times.

This technique has been applied to accurately measure the diffusion coefficient of the enhanced green fluorescent protein (EGFP) in the cytoplasm of living *Escherichia coli* [16].

### 2.3. 4Pi Microscopy

New light microscopy concepts have been developed to improve the spatial resolution up to about 20 nm [17]. Among them, 4Pi microscopy takes advantage of two opposite microscope objectives with high numerical apertures [18]. Its principle is illustrated in Figure 3. Coherent light from a laser is split into two beams, which are focused at the same point onto a sample by two opposite objectives. Constructive interference of the two beams enhances the focusing of the light, and the illuminated region is narrower along the optical axis than in the case of the common confocal microscope. In 4Pi microscopy, various types of illumination and detection are utilized: type A corresponds to the illumination via two objectives with constructive interference and detection through one of the objectives in a confocal mode (commercially available); in type C, both illumination and detection are performed using two objectives, with constructive interference in both cases.

Increasing the number of objectives to two (or more) decreases the detection volume and increases the fluorescence collection efficiency. The 4Pi microscope features a point spread function with a central peak of 100 nm width in the axial direction and 220 nm width in the focal plane. In addition, the point spread function has smaller secondary maxima spaced on the optical axis at a distance of about half a wavelength from the main maximum. The essential point is that about 90% of the fluorescence signal stems from the main peak. Thus, the observation volume is virtually coincident with that of the main maximum of the point spread function.

A major problem in the adaptation of 4Pi microscopy to FCS is to account for the complicated point spread function of the microscope. A theoretical and computational framework has therefore been developed for data analysis, and validated by measurements on model systems [19].

### 2.4. Stimulated Emission Depletion Microscopy: STED

A different approach to overcome the diffraction barrier is to use stimulated emission depletion (STED) of the fluorescent molecular state (Figure 3) [17]. STED is a far-field method bearing sub-diffraction analysis volumes suitable for FCS. In STED, a regular diffraction-limited focal spot (green) is used to excite the fluorescence, while a second laser beam (red) stimulates the excited molecules down to their ground state. The laser beam for stimulated emission is custom-tailored to feature a zero-intensity minimum at the center but high intensity in the focal periphery. This configuration ensures that fluorescence occurs only in the very center of the focal spots and is strongly suppressed in the spots periphery. An additional attractive feature of STED is that it allows to adjust the detection volume by increasing the power of the stimulating beam.

The first implementation of a STED experiment with FCS was published by Kastrup *et al.* [20]. In a series of FCS measurements on a dilute solution of a red-fluorescing oxazine dye, the STED irradiance was successively increased yielding a 25-fold reduction of the axial diffusion time, equivalent to a 5-fold reduction of the focal volume. However, much stronger analysis volume reduction can be expected with that method.

In a second contribution, STED-FCS was used to investigate the cell membrane architecture at the nanoscale [21]. Single diffusing lipid molecules were detected in nanosized areas in the plasma membrane of living cells. Tuning of the probed area 70-fold below the diffraction barrier reveals that sphingolipids and glycosylphosphatidylinositol-anchored proteins are transiently trapped in cholesterol-mediated molecular complexes dwelling within 20 nm diameter areas. This tunable noninvasive optical recording combined to nanoscale imaging is a powerful new approach to study the dynamics of molecules in living cells.

## 3. Improved Single Molecule Fluorescence Detection by Using Photonic Structures

**Figure 4.**
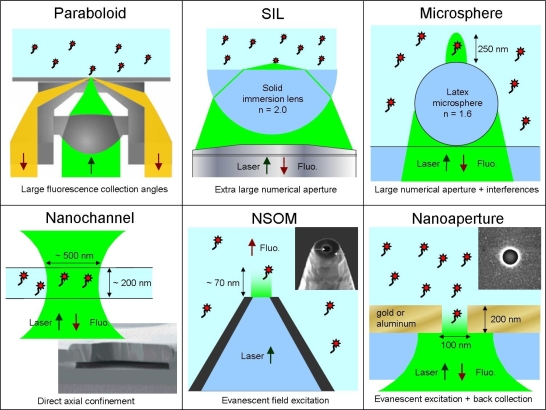
Designs for improved single molecule fluorescence detection by using photonic structures; see text for details.

Optical methods of this section introduce photonic structures from millimeter to nanometer size to improve the detection of the fluorescence signal. The first two techniques (paraboloid collector and solid immersion lens) make the transition with the methods discussed in Section 2 and introduce many key concepts of nanophotonics. The last four techniques go really into the nanoworld of photonics. In addition to Figure 3, Figure 4 describes the photonic methods of this section for enhanced detection.

### 3.1. Paraboloid Collector

This main core of this technique is to replace the microscope objective by a paraboloid glass segment acting as a mirror for collecting the fluorescence (Figure 4). A special feature of the system is its ability to sample not only fluorescence that is emitted below the angle of total internal reflection (known as the critical angle) but also particularly the light above the critical angle. This is especially advantageous for collecting the fluorescence of surface-bound molecules. This specific optical system leads to a fluorescence collection efficiency to more than 65% of the total of emitted light, whereas high numerical aperture microscope objectives are able to collect 44% at best. Moreover, the detection volume can be restricted ten times below the standard confocal volume [22, 23]. This allows for a strong discrimination of bulk-generated against surface-generated fluorescence, which may be of great value when surface-binding processes are monitored.

The potential of a paraboloid collector (also referred to as supercritical angle objective) for FCS has been discussed in [24], demonstrating a clear advantage of paraboloid-FCS for diffusion measurements in lipid membranes. This method circumvents the need to illuminate at large angles as with TIRF and achieves an excellent axial confinement in combination with a small lateral excitation spot of a customized confocal microscope.

### 3.2. Solid Immersion Lens: SIL

For FCS, confinement of the excitation field and high fluorescence collection efficiency are key parameters. Both are directly proportional to the numerical aperture (NA) of the microscope objective system. Immersing the analysis volume with a liquid of a high refractive index, e.g., oil, increases the effective NA. Another way to increase the NA can be achieved by utilizing a solid immersion lens or SIL (Figure 4). A SIL is generally a hemisphere made in a material of high refractive index that is set at the focus of a microscope objective to further increase the overall NA of the whole system [25].

To perform FCS at high molecular concentrations with an ultra-high NA, an option is to combine a 0.6 NA air objective to a solid immersion lens (SIL) with refractive index close to 2 [26]. Experiments performed on a commercial FCS apparatus (Zeiss ConfoCorr 1) shown 50% higher collection efficiency and better field confinement for the SIL system in comparison to the conventional confocal set-up. Performances can be increased further by aberration compensation and pre-shaping the incident wavefront to obtain near diffraction-limited performance [27].

### 3.3. Microspheres

Dielectric microspheres can be a viable alternative for enhanced fluorescence detection in solution, offering a simple and low-cost method [28]. When a latex microsphere is illuminated with a tightly-focused Gaussian beam, it over-focuses light in a region with subwavelength dimensions in both the transverse and longitudinal directions, creating high local intensities. This effect stems from interferences between the field scattered by the sphere and the high angular components of the incident Gaussian beam passing aside the sphere [29].

Latex microspheres have been exploited to enhance the detection of single fluorescent molecules in FCS, yielding a simultaneous decrease of the confocal observation volume by an order of magnitude and an enhancement of the fluorescence brightness by a factor of five [28]. This phenomenon was explained as the microsphere increases the excitation intensity sensed by the molecule up to a factor of 2.2, while at the same time it allows for a gain in collection efficiency up to 60% by redirecting the light emitted at large incidences towards the optical axis [30]. Commercially available latex microspheres therefore appear as an attractive and cost-effective route to enhance the confocal microscopes for FCS without requiring expensive nanofabrication facilities.

Latex microspheres can also be combined with a simple low NA lens to form a high performance disposable optical system. This offers a simple and low-cost alternative to the expensive microscope objectives used in FCS [31]. Moreover, microspheres can be combined to an optical fiber for remote FCS analysis in an endoscopic apparatus [32]. The technique is sensitive enough to detect single fluorescent molecules. This offers new opportunities for reducing the bulky microscope setup and extending FCS to remote or in vivo applications.

### 3.4. Nanofluidic Channels

Novel nanofabrication techniques enable the realization of structures to confine the sampling volume in the fluorescent solution on sizes much smaller than the diffraction limit. Mechanically restricting the observation volume directly improves the efficiency of FCS toward micromolar concentration ranges [33]. It also simultaneously improves slightly the fluorescence rate per molecule by detecting only the molecules that sense the strongest excitation intensity at the center of the laser spot.

Foquet and coworkers have used nanofabricated channels with dimensions smaller or equal to the width and depth of a diffraction-limited detection volume in confocal microscopy for FCS studies [34, 35]. An effective detection volume of few tens of attoliters was achieved (see Figure 4). The nanochannels had typical dimensions of a few hundred nanometers in their cross-section. Such nanometer sized channels offer natural platforms for optofluidic sensing devices to achieve high-throughput analysis systems at the single molecule level. Furthermore, FCS can be applied successfully to measure velocities and directionalities in different capillaries for hydrodynamic and electrophoretic flows [36].

Lastly, nanochannels open new opportunities for full implementation of FCS on a chip [37]. Full planar integration can be achieved by lithographic definition of sub-picoliter excitation volumes using intersecting solid and liquid-core optical waveguides. Silicon micro and nanophotonics are thus to replace free-space microscopes, and offer even higher performances. This permits implementation of numerous diagnostic applications on compact planar optofluidic devices.

### 3.5. Near-Field Scanning Optical Microscope: NSOM

Near-field scanning optical microscopy (NSOM) is based on a subwavelength-sized light source that is raster-scanned across a surface at a distance of a few nanometers to image the sample. Near-field optical methods have been widely developed to provide spatial resolutions of 10?30 nm [38]. Compared to the other systems described in this section, NSOM bears the essential ability to image the sample.

NSOM microscopes are typically implemented using tapered optical single-mode fibers that are coated with a thin layer of aluminum. At the apex of the tip, an aperture of nanometer size is opened by focused ion beam milling (Figure 4). The region illuminated at the apex of the tip is constrained both in the lateral and longitudinal directions (for an aperture diameter typically below half the wavelength, the laser field evanescently decays inside the aperture, with a longitudinal extend of about 100 nm).

FCS measurements using NSOM probes have been reported in [39]. An order of magnitude reduction in the area compared to confocal FCS has been achieved, while the use of probes with smaller apertures is expected to provide an additional order of magnitude reduction. It was also reported that bare chemically etched, tapered fiber tips could be implemented for NSOM-FCS [40].

Very recently, NSOM-FCS was applied to study proteins diffusion passing through individual nuclear pore complexes of the nuclear envelope [41]. FCS characterizes the translocation as driven by Brownian motion and determines the related kinetic constants.

### 3.6. Nanometric Apertures

A simple and elegant way of generating a reduced analysis volume for FCS implements a single nanometric aperture milled in an opaque metallic film. As illustrated on Figure 4, the nanoaperture acts as a pinhole filter located directly into the object plane. When the aperture diameter is sufficiently reduced below the cut-off diameter of the fundamental excitation mode that may propagate through the aperture, the light inside the aperture is confined to a rapidly decaying evanescent mode, with a decay length of a few tens of nanometers (such devices have thus been named zero-mode waveguides). This concepts bears strong relations with NSOM probes, yet in a much more robust and easy to use configuration. Since both excitation and fluorescence collection are performed from the glass substrate side, very high detection efficiencies can be reached.

An impressive amount of literature has been published about the use of nanoapertures for FCS, since the pioneering demonstration of Levene and coworkers in 2003 [42]. With aperture diameters down to 30 nm, detection volumes of a few tens of zeptoliters (1 zL = 10^21^ L) have been generated, which are about four orders of magnitude smaller than diffraction-limited confocal volumes. DNA polymerase activity can be monitored at the single molecule level at dye concentrations of a few tens of micromolar. Implementation of nanoapertures currently provides the best performances for FCS at high concentrations [43, 44]. The applications can be extended to dual-color cross-correlation FCCS analysis [45], and to monitor flow mixing [46].

A second major effect brought by the sub-wavelength aperture is that it can significantly enhance the detected fluorescence rate per emitter. Using single rhodamine 6G molecules in isolated 150 nm diameter apertures milled in an aluminum film, a 6.5 fold enhancement of the fluorescence rate per molecule was reported as compared to free solution [47, 48]. Further enhancement up to 25-fold can be obtained by tuning the plasmon properties of the nanoapertures [49–51]. This provides a dramatic increase in signal-to-noise ratio (SNR) for FCS even at single molecule resolution. An experimental gain in SNR of about 1 order of magnitude, corresponds to a 100-fold reduction of the experiment duration, evidencing the feasibility of FCS analysis with fast integration times of about 1 s [52]. This opens the way to monitoring of a variety of biochemical reactions at reduced time scales.

Biophotonic applications of nanoapertures are reviewed for instance in [53]. The illuminated area well below the diffraction limit has proven of essential interest for FCS studies in lipid bilayers [54, 55] as well as in live cell membranes [56–58]. Nanoapertures combined to FCS offer the advantages of both high spatial and temporal resolution together with a direct statistical analysis.

Lastly, a very promising application of nanometric apertures concerns real-time single-molecule DNA sequencing [59]. Performing high-throughput, high-accuracy DNA sequencing at low costs has become a major issue, largely attracted by the growing potential of quantitative genomics. Each nanoaperture forms a nano-observation chamber for watching the activity of a single DNA polymerase enzyme performing DNA sequencing by synthesis.

## 4. Conclusions

**Figure 5.**
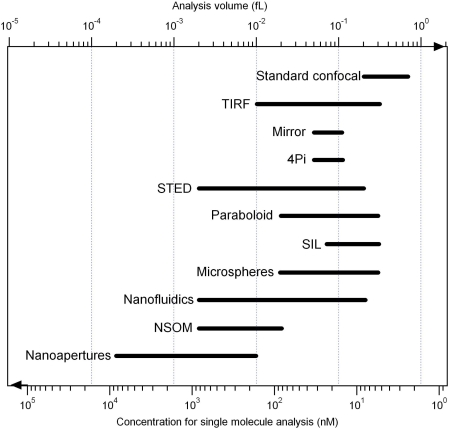
Range for analysis volumes and corresponding concentrations to ensure one single molecule in the analysis volume for the different schemes discussed here.

At least ten different strategies have been demonstrated to enhance single molecule detection techniques beyond the limits set by optical diffraction. Table 1 summarizes their respective properties for reducing the analysis volume and enhancing the detected fluorescence rate per molecule. This enables fast comparison between the techniques and standard confocal microscopy. Table 1 also provides our arbitrary vision of the respective complexity required while setting up these novel strategies. Lastly, Figure 5 illustrates the practical ranges of concentrations offered by each method for single molecule detection.

Based on the end-user application requirements, we believe that many photonic strategies exist to enhance single molecule fluorescence detection. For those needing simple and robust integration with commercially available systems, TIRF microscope objectives, paraboloid objectives and polystyrene microspheres offer relevant improvements. For maximum integration into optofluidic systems, nanochannels possibly equipped with microspheres or nanoapertures are the natural choice. Lastly, for maximum performance, nanoapertures offer the best gains, both in volume reduction and fluorescence rate enhancement.

## Figures and Tables

**Table 1.** Summary of the two main characteristics for single molecule detection systems: volume reduction and fluorescence signal enhancement factors as compared to state-of-the-art confocal microscope.

Type	Volume reduction	Fluo. enhancement	Complexity

Confocal	x1	x1	*

TIRF	x10	x2	*
Mirror	x6	x4	*
4Pi	x6	x4	***
STED	x100	?	***

Paraboloid	x10	x2	*
SIL	x3	x1.5	**
Microsphere	x10	x5	*
Nanofluidics	x100	x3	**
NSOM	x100	?	***
Nanoapertures	x1000	x25	**

## References

[1] Zander C Enderlein J Keller RA Single-Molecule Detection in Solution—Methods and Applications VCH-Wiley Berlin, Germany 2002

[2] Craighead HG Future lab-on-a-chip technologies for interrogating individual molecules Nature 2006 442 387 393 16871206 10.1038/nature05061

[3] Maiti S Haupts U Webb WW Fluorescence correlation spectroscopy: diagnostics for sparse molecules Proc. Nat. Acad. Sci. USA 1997 94 11753 11757 9342306 10.1073/pnas.94.22.11753 PMC33774

[4] Webb WW Fluorescence correlation spectroscopy: Inception, biophysical experimentations, and prospectus Appl. Opt 2001 40 3969 3983 18360431 10.1364/ao.40.003969

[5] Briddon SJ Hill SJ Pharmacology under the microscope: The use of fluorescence correlation spectroscopy to determine the properties of ligandreceptor complexes Trends Pharmacol. Sci 2007 28 637 645 18001848 10.1016/j.tips.2007.09.008 PMC2148440

[6] Huser T Nano-Biophotonics: New tools for chemical nano-analytics Curr. Opin. Chem. Biol 2008 12 497 504 18786651 10.1016/j.cbpa.2008.08.012 PMC2652705

[7] Blom H Kastrup L Eggeling C Fluorescence fluctuation spectroscopy in reduced detection Volumes Curr. Pharm. Biotechnol 2006 7 51 66 16472133 10.2174/138920106775789629

[8] Mannion JT Craighead HG Nanofluidic structures for single biomolecule fluorescent detection Biopolymers 2006 85 131 143 17103421 10.1002/bip.20629

[9] Thompson NL Burghardt TP Axelrod D Measuring surface dynamics of biomolecules by total internal–Reflection fluorescence with photobleaching recovery or correlation spectroscopy Biophys. J 1981 33 435 454 7225515 10.1016/S0006-3495(81)84905-3 PMC1327440

[10] Hassler K Leutenegger M Rigler P Rao R Rigler R Gösch M Lasser T Total internal reflection fluorescence correlation spectroscopy (TIR-FCS) with low background and high count-rate per molecule Opt. Express 2005 13 7415 7423 19498766 10.1364/opex.13.007415

[11] Starr TE Thompson NL Total internal reflection with fluorescence correlation spectroscopy: Combined surface reaction and solution diffusion Biophys. J 2001 80 1575 1584 11222318 10.1016/S0006-3495(01)76130-9 PMC1301349

[12] Borejdo J Calander N Gryczynski Z Gryczynski I Fluorescence correlation spectroscopy in surface plasmon coupled emission microscope Opt. Express 2006 14 7878 7888 19529155 10.1364/oe.14.007878

[13] Leutenegger M Blom H Widengren J Eggeling C Gösch M Leitgeb RA Lasser T Dual-color total internal reflection fluorescence cross-correlation spectroscopy J Biomed Optics 2006 11 040502doi:10.1117/1.2221714 10.1117/1.2221714 16965125

[14] Lenne PF Etienne E Rigneault H Subwavelength patterns and high detection efficiency in fluorescence correlation spectroscopy using photonic structures Appl. Phys. Lett 2002 80 4106 4108

[15] Rigneault H Lenne PF Fluorescence correlation spectroscopy on a mirror J. Opt. Soc. Am. B 2003 20 2203 2214 10.1364/ao.45.004497 16778960

[16] Etienne E Lenne PF Sturgis JN Rigneault H Confined diffusion in tubular structures analyzed by fluorescence correlation spectroscopy on a mirror Appl. Opt 2006 45 4497 4507 16778960 10.1364/ao.45.004497

[17] Hell SW Toward fluorescence nanoscopy Nat. Biotechnol 2003 21 1347 1355 14595362 10.1038/nbt895

[18] Hell SW Stelzer EHK Fundamental improvement of resolution with a 4Pi-confocal fluorescence microscope using twophoton excitation Opt. Commun 1992 93 277 282

[19] Arkhipov A Hüve J Kahms M Peters R Schulten K Continuous fluorescence microphotolysis and correlation spectroscopy using 4Pi microscopy Biophys. J 2007 93 4006 4017 17704168 10.1529/biophysj.107.107805 PMC2084225

[20] Kastrup L Blom H Eggeling C Hell SW Fluorescence fluctuation spectroscopy in subdiffraction focal volumes Phys Rev Lett 2005 94 178104:1 178104:4 15904340 10.1103/PhysRevLett.94.178104

[21] Eggeling C Ringemann C Medda R Schwarzmann G Sandhoff K Polyakova S Belov VN Hein B von Middendorff C Schönle A Hell SW Direct observation of the nanoscale dynamics of membrane lipids in a living cell Nature 2008 457 1159 1162 19098897 10.1038/nature07596

[22] Enderlein J Ruckstuhl T Seeger S Highly efficient optical detection of surface-generated fluorescence Appl. Opt 1999 38 724 732 18305670 10.1364/ao.38.000724

[23] Ruckstuhl T Enderlein J Jung S Seeger S Forbidden light detection from single molecules Anal. Chem 2000 72 2117 2123 10815974 10.1021/ac991358k

[24] Ries J Ruckstuhl T Verdes D Schwille P Supercritical angle fluorescence correlation spectroscopy Biophys. J 2008 94 221 229 17827221 10.1529/biophysj.107.115998 PMC2134864

[25] Koyama K Yoshita M Baba M Suemoto T Akiyama H High collection efficiency in fluorescence microscopy with a Solid Immersion Lens Appl. Phys. Lett 1999 75 1667 1669

[26] Serov A Rao R Gösch M Anhut T Martin D Brunner R Rigler R Lasser T High light field confinement for fluorescent correlation spectroscopy using a solid immersion lens Biosens. Bioelectron 2004 20 431 435 15494221 10.1016/j.bios.2004.02.028

[27] Rao R Mitic J Serov A Leitgeb RA Lasser T Field confinement with aberration correction for solid immersion lens based fluorescence correlation spectroscopy Opt. Commun 2007 271 462 469

[28] Gérard D Wenger J Devilez A Gachet D Stout B Bonod N Popov E Rigneault H Strong electromagnetic confinement near dielectric microspheres to enhance single-molecule fluorescence Opt. Express 2008 16 15297 15303 18795067 10.1364/oe.16.015297

[29] Devilez A Bonod N Stout B Gérard D Wenger J Rigneault H Popov E Three-dimensional subwavelength confinement of photonic nanojets Opt. Express 2009 17 2089 2094 19219113 10.1364/oe.17.002089

[30] Gérard D Devilez A Aouani H Stout B Bonod N Wenger J Popov E Rigneault H Efficient excitation and collection of single molecule fluorescence close to a dielectric microsphere J. Opt. Soc. Am. B 2009 26 1473 1478

[31] Wenger J Gérard D Aouani H Rigneault H Disposable microscope objective lenses for fluorescence correlation spectroscopy using latex microspheres Anal. Chem 2008 80 6800 6804 18681458 10.1021/ac801016z

[32] Aouani H Deiss F Wenger J Ferrand P Sojic N Rigneault H Optical-fiber-microsphere for remote fluorescence correlation spectroscopy Opt. Express 2009 17 18912 18919 10.1364/OE.17.019085 20372645

[33] Brinkmeier M Dijrre K Riebeseel K Rigler R Confocal spectroscopy in microstructures Biophys. Chem 1997 66 229 239 17029876 10.1016/s0301-4622(97)00065-3

[34] Foquet M Korlach J Zipfel WR Webb WW Craighead HG DNA fragment sizing by single molecule detection in submicrometer-sized closed fluidic channels Anal. Chem 2002 74 1415 1422 11922312 10.1021/ac011076w

[35] Foquet M Korlach J Zipfel WR Webb WW Craighead HG Focal volume confinement by submicrometer-sized fluidic channels Anal. Chem 2004 76 1618 1626 15018559 10.1021/ac035088o

[36] Lenne PF Colombo D Giovannini H Rigneault H Flow profiles and directionality in microcapillaries measured by fluorescence correlation spectroscopy Single Mol 2002 3 194 200

[37] Yin D Lunt EJ Barman A Hawkins AR Schmidt H Microphotonic control of single molecule fluorescence correlation spectroscopy using planar optofluidics Opt. Express 2007 15 7290 7295 19547052 10.1364/oe.15.007290

[38] Lewis A Taha H Strinkovski A Menevitch A Katchatouriants A Dekhter R Amman E Near-field optics: From subwavelength illumination to nanometric shadowing Nat. Biotechnol 2003 21 1378 1386 14595366 10.1038/nbt898

[39] Vobornik D Banks DS Lu Z Fradin C Taylor R Johnston LJ Fluorescence correlation spectroscopy with sub-diffraction-limited resolution using near-field optical probes Appl Phys Lett 2008 93 163904:1 163904:3

[40] Lu G Lei FH Angiboust JF Manfait M Confined detection volume of fluorescence correlation spectroscopy by bare fiber probes Eur Biophys J 2009doi:10.1007/s00249-009-0508-z 10.1007/s00249-009-0508-z 19575194

[41] Herrmann M Neuberth N Wissler J Pérez J Gradl D Naber A Near-field optical study of protein transport kinetics at a single nuclear pore Nano Lett 2009 9 3330 3336 19591452 10.1021/nl901598z

[42] Levene MJ Korlach J Turner SW Foquet M Craighead HG Webb WW Zero-mode waveguides for single-molecule analysis at high concentrations Science 2003 299 682 686 12560545 10.1126/science.1079700

[43] Samiee KT Foquet M Guo L Cox EC Craighead HG Lambda repressor oligomerization kinetics at high concentrations using fluorescence correlation spectroscopy in zero-mode waveguides Biophys. J 2005 88 2145 2153 15613638 10.1529/biophysj.104.052795 PMC1305266

[44] Leutenegger M Gösch M Perentes A Hoffmann P Martin OJF Lasser T Confining the sampling volume for Fluorescence Correlation Spectroscopy using a sub-wavelength sized aperture Opt. Express 2006 14 956 969 19503416 10.1364/opex.14.000956

[45] Wenger J Gérard D Lenne PF Rigneault H Dintinger J Ebbesen TW Boned A Conchonaud F Marguet D Dual-color fluorescence cross-correlation spectroscopy in a single nanoaperture: Towards rapid multicomponent screening at high concentrations Opt. Express 2006 14 12206 12216 19529650 10.1364/oe.14.012206

[46] Liao D Galajda P Riehn R Ilic R Puchalla JL Yu HG Craighead HG Austin RH Single molecule correlation spectroscopy in continuous flow mixers with zero-mode waveguides Opt. Express 2008 16 10077 10090 18607415 10.1364/oe.16.010077

[47] Rigneault H Capoulade J Dintinger J Wenger J Bonod N Popov E Ebbesen TW Lenne PF Enhancement of single-molecule fluorescence detection in subwavelength apertures Phys Rev Lett 2005 95 117401:1 117401:4 16197045 10.1103/PhysRevLett.95.117401

[48] Wenger J Lenne PF Popov E Rigneault H Dintinger J Ebbesen TW Single molecule fluorescence in rectangular nano-apertures Opt. Express 2005 13 7035 7044 19498725 10.1364/opex.13.007035

[49] Wenger J Gérard D Bonod N Popov E Rigneault H Dintinger J Mahboub O Ebbesen TW Emission and excitation contributions to enhanced single molecule fluorescence by gold nanometric apertures Opt. Express 2008 16 3008 3020 18542387 10.1364/oe.16.003008

[50] Gérard D Wenger J Bonod N Popov E Rigneault H Mahdavi F Blair S Dintinger J Ebbesen TW Nanoaperture-enhanced fluorescence: Towards higher detection rates with plasmonic metals Phys Rev B 2008 77 045413doi:10.1103/PhysRevB.77.045413

[51] Aouani H Wenger J Gérard D Rigneault H Devaux E Ebbesen TW Mahdavi F Xu T Blair S Crucial role of the adhesion layer on the plasmonic fluorescence enhancement ACS Nano 2009 3 2043 2048 10.1021/nn900460t 19518085

[52] Wenger J Gérard D Aouani H Rigneault H Lowder B Blair S Devaux E Ebbesen TW Nanoaperture-enhanced signal-to-noise ratio in fluorescence correlation spectroscopy Anal. Chem 2009 81 834 839 19099408 10.1021/ac8024015

[53] Lenne PF Rigneault H Marguet D Wenger J Fluorescence fluctuations analysis in nanoapertures: Physical concepts and biological applications Histochem. Cell Biol 2008 130 795 805 18800223 10.1007/s00418-008-0507-7

[54] Samiee KT Moran-Mirabal JM Cheung YK Craighead HG Zero mode waveguides for single-molecule spectroscopy on lipid membranes Biophys. J 2006 90 3288 3299 16461393 10.1529/biophysj.105.072819 PMC1432119

[55] Wenger J Rigneault H Dintinger J Marguet D Lenne PF Single-fluorophore diffusion in a lipid membrane over a subwavelength aperture J Biol Phys 2006 32 SN1 SN4 19669430 10.1007/s10867-006-2909-x PMC3022499

[56] Edel JB Wu M Baird B Craighead HG High spatial resolution observation of single molecule dynamics in living cell membranes Biophys. J 2005 88 L43 L45 15821167 10.1529/biophysj.105.061937 PMC1305672

[57] Wenger J Conchonaud F Dintinger J Wawrezinieck L Ebbesen TW Rigneault H Marguet D Lenne PF Diffusion analysis within single nanometric apertures reveals the ultrafine cell membrane organization Biophys. J 2007 92 913 919 17085499 10.1529/biophysj.106.096586 PMC1779989

[58] Moran-Mirabal JM Torres AJ Samiee KT Baird B Craighead HG Cell investigation of nanostructures: Zero-mode waveguides for plasma membrane studies with single molecule resolution Nanotechnology 2007 18 195101:1 195101:10

[59] Eid J Fehr A Gray J Luong K Lyle J Otto G Peluso P Rank D Baybayan P Bettman B Bibillo A Bjornson K Chaudhuri B Christians F Cicero R Clark S Dalal R deWinter A Dixon J Foquet M Gaertner A Hardenbol P Heiner C Hester K Holden D Kearns G Kong X Kuse R Lacroix Y Lin S Lundquist P Ma C Marks P Maxham M Murphy D Park I Pham T Phillips M Roy J Sebra R Shen G Sorenson J Tomaney A Travers K Trulson M Vieceli J Wegener J Wu D Yang A Zaccarin D Zhao P Zhong F Korlach J Turner S Real-time DNA sequencing from single polymerase molecules Science 2009 323 133 138 19023044 10.1126/science.1162986

